# Complete Genome Characterization of Eight Human Parainfluenza Viruses from the Netherlands

**DOI:** 10.1128/MRA.00125-19

**Published:** 2019-04-11

**Authors:** My V. T. Phan, Georgina Arron, Corine H. GeurtsvanKessel, Robin C. Huisman, Richard Molenkamp, Marion P. G. Koopmans, Matthew Cotten

**Affiliations:** a Department of Viroscience, Erasmus MC, Rotterdam, the Netherlands; Loyola University Chicago

## Abstract

We report the complete genome sequences of eight human parainfluenza viruses (HPIV) belonging to Human respirovirus 1 (HPIV-1), Human respirovirus 3 (HPIV-3), Human rubulavirus 2 (HPIV-2), and Human rubulavirus 4 (HPIV-4). The genome sequences were generated using random-primed next-generation sequencing and represent the first HPIV full-genome sequences from the Netherlands.

## ANNOUNCEMENT

The human parainfluenza viruses (HPIVs) are members of the Paramyxoviridae family of viruses and are a common cause of acute upper and lower respiratory infections. Although HPIV infections are generally mild and self-limiting, severe infections leading to hospitalization may occur, particularly in infants, young children, and immunocompromised individuals (1).

The HPIV virion encloses a single-stranded negative-sense RNA genome with a length of ∼15,000 nucleotides (nt), encoding the following 6 structural proteins: the nucleoprotein (NP), phosphoprotein (P), matrix protein (M), fusion protein (F), hemagglutinin-neuraminidase (HN), and large protein (L). HPIVs are subdivided into 2 genera, Respirovirus (Human respirovirus 1 [HPIV-1] and Human respirovirus 3 [HPIV-3]) and Rubulavirus (Human rubulavirus 2 [HPIV-2] and Human rubulavirus 4 [HPIV-4]). In the Netherlands, HPIVs were the third most common viral pathogen in noninfluenza respiratory infections (after rhinovirus and adenovirus) from 2006 to 2015, according to a National Institute for Public Health and the Environment (RIVM) report (2). Surprisingly, given the clinical frequency of HPIVs, as of 22 January 2019, there were no HPIV full genomes from the Netherlands identified in GenBank, leading to a knowledge gap in the local virus diversity. Also, given the importance of nucleic acid-based diagnostics, documenting local viral sequences is essential for maintaining sensitive clinical diagnostics capable of detecting locally circulating HPIV strains.

Eight samples from the years 2013 to 2016 that were positive for HPIV (HPIV-1, HPIV-2, HPIV-3, and HPIV-4) were randomly chosen (Table 1). The HPIVs were isolated from respiratory patients (youngest, 2 months of age, and oldest, 63 years of age) and propagated in LLC-MK2 (ATCC CCL-7, *Macaca mulatta*, monkey, rhesus) cell lines. Passage 3 of each sample, except that of sample t146a305 (for which passage 2 was used), was subjected to real-time PCR (3) to confirm the presence of HPIV and then used as follows for direct sequencing. Total viral nucleic acid was extracted from the 8 culture supernatants using a High Pure viral RNA extraction kit (Roche, Mannheim, Germany) following the manufacturer’s instructions. Extracted RNA was reverse transcribed using random hexamers that avoid rRNA (4), followed by second-strand synthesis using Klenow fragments (New England Biolabs), as previously described (5). The resulting double-stranded DNA (dsDNA) was used to prepare for sequencing libraries using an Ion Xpress Plus fragment library kit (part number 4471269) and subsequently sequenced on the Ion Torrent S5XL platform to generate 2.3 × 10^6^ to 5.1 × 10^6^ reads per sample (median read length, 275 to 300 nt). Raw reads were trimmed from the 3′ end to a median Phred score of 25 and minimum length of 75 nt using QUASR (6) and *de novo* assembled using SPAdes version 3.13.0 (7). In all samples, 1 to 2 contigs spanning the full genome were obtained; subgenomic contigs were combined using Geneious (version 9.1.8).

**TABLE 1:** Clinical and sequence data for 8 HPIV samples

Sample identifier	GenBank accession no.	SRR accession no.	BioSample no.	Genome GC content (%)	Species	HPIV genotype	Date of original sample (mo/day/yr)	Patient age	Clinical symptom(s)^*c*^	Passage no. (LLC-MK2)	Cell culture *C_T_* value^*d*^	Genome length (nt)	No. of mapped reads^*a*^	Total no. of reads	Avg coverage (×)^*b*^
t146a290	MH892403	SRR8512262	SAMN10838957	37.2	*Human respirovirus 1*	HPIV-1	10/28/2013	9 mos	Respiratory insufficiency	3	16.7	15,675	425,854	1,927,124	6,792
t146a291	MH892404	SRR8512263	SAMN10838958	37.2	*Human respirovirus 1*	HPIV-1	2/5/2013	2 yrs	Pierre Robin syndrome, cleft, respiratory insufficiency, tracheal cannula	3	19	15,422	17,598	2,024,742	285
t146a292	MH892405	SRR8512264	SAMN10838959	38.4	*Human rubulavirus 2*	HPIV-2	12/15/2014	6 yrs	ILD, fever, and coughing	3	12.3	15,684	18,263	1,828,911	291
t146a293	MH892406	SRR8512265	SAMN10838960	38.7	*Human rubulavirus 2*	HPIV-2	4/2/2014	43 yrs	Increasing dyspnea, mucosal swelling, stridor, hoarseness	3	11	15,654	264,338	1,939,223	4,222
t146a304	MH892409	SRR8512258	SAMN10838961	35.1	*Human respirovirus 3*	HPIV-3	8/1/2016	63 yrs	Respiratory insufficiency	3	10.6	15,387	1,355,991	4,099,107	22,031
t146a305	MH892410	SRR8512259	SAMN10838962	35.2	*Human respirovirus 3*	HPIV-3	11/29/2013	14 yrs	Fever, mucus	2	14	15,409	330,469	2,589,260	5,362
t146a296	MH892407	SRR8512260	SAMN10838963	36.3	*Human rubulavirus 4*	HPIV-4	11/8/2013	2 mos	Respiratory insufficiency	3	11.5	17,079	46,139	1,743,284	675
t146a303	MH892408	SRR8512261	SAMN10838964	36.3	*Human rubulavirus 4*	HPIV-4	9/13/2013	8 mos	Tracheal cannula, URI	3	13.4	17,031	21,081	2,598,198	309

a Total number of quality-controlled reads mapped to final genome.

b Number of mapped reads times 250 divided by the length of the genome.

c ILD, interstitial lung disease; URI, upper respiratory infection.

d *C_T_*, threshold cycle.

Eight complete HPIV genomes were assembled from short-read data, and their open reading frames (ORFs) were checked to ensure intact ORFs. Results from BLAST searches of these genomes showed that they share 98% to 99% similarity at the nucleotide level with contemporary strains from Thailand (2012, GenBank accession number KM190940) and France (2009, accession number KF687313) for HPIV-1, from the United States (2016, accession number KY674949, and 2017, accession number MF077312) for HPIV-2, from the United States (2015, accession number KY973558) and France (2009, accession number KF530233) for HPIV-3, and from Taiwan (2010, accession number KY460518) for HPIV-4. Annotation for the open reading frames was performed in Geneious using information in the GenBank entries for the four reference genomes listed under the accession numbers NC_003461 (HPIV-1), NC_001796 (HPIV-3), NC_003443 (HPIV-2), and NC_021928 (HPIV-4).

In conclusion, we report here the first 8 genomes of HPIV isolated from the Netherlands.

### Data availability.

The eight HPIV genomic sequences described in this study have been deposited in GenBank under the accession numbers MH892403 to MH892410, with the BioSample and run accession numbers shown in Table 1. The corresponding short-read data are available in the SRA under the BioProject accession number PRJNA517593.
